# Funding the future: safeguarding pediatric health equity through CMS and CHIP

**DOI:** 10.3389/fpubh.2025.1611720

**Published:** 2025-07-24

**Authors:** Carlie N. Myers, Meera Rao, Rishiraj Bandi, Sebastian Densley, Daniella Diaz, Michelle Knecht, Lea Sacca

**Affiliations:** ^1^Cincinnati Children's Hospital Medical Center, University of Cincinnati College of Medicine Cincinnati, Cincinnati, OH, United States; ^2^Charles E. Schmidt College of Medicine, Florida Atlantic University, Boca Raton, FL, United States; ^3^Charles E. Schmidt College of Science, Florida Atlantic University, Boca Raton, FL, United States

**Keywords:** CHIP, medicare, CMS, public insurance, pediatric, health disparities, health inequities

## Abstract

Pediatric health disparities remain widespread among marginalized populations, driven by structural racism, poverty, and unequal access to care. While the Centers for Medicare and Medicaid Services (CMS) and the Children's Health Insurance Program (CHIP) have advanced equity in pediatric healthcare, ongoing threats to federal funding jeopardize this progress. This commentary examines five articles, including four CMS-funded interventions that address pediatric health inequities by targeting key social determinants of health (SDoH), including access to care, neighborhood conditions, and insurance coverage. Interventions reviewed include Functional Family Therapy (FFT) for adjudicated youth, a hospital-led asthma management initiative in Ohio, community-based care engagement strategies in disorganized Chicago neighborhoods, and a national policy analysis of CHIP's effectiveness. Across these studies, common themes emerged: community engagement, cross-sector collaboration, and expanded insurance access improved outcomes and reduced costs. Medicaid expansion reduced in-hospital mortality and improved access to rehabilitative care, while CHIP increased preventive service use among near-poor children. However, persistent barriers—including racial and geographic inequities—continue to limit care engagement. While pediatric healthcare research has moved beyond disparity detection, implementation of targeted, evidence-based interventions remains limited. Without sustained investment in CMS and CHIP, the infrastructure supporting equitable pediatric care may erode, exacerbating health gaps for the most vulnerable. Policymakers must prioritize funding and support for initiatives that integrate medical, social, and structural solutions to pediatric health disparities. Strengthening CMS-supported programs is essential not only for improving child health outcomes but also for reducing long-term healthcare costs and advancing pediatric health equity.

## Introduction

Healthcare disparities remain pervasive among marginalized and minoritized pediatric populations, contributing to inequitable health outcomes (1). Recognizing its role in addressing these disparities, the Centers for Medicare and Medicaid Services (CMS) has increasingly focused on equitable pediatric healthcare delivery (2, 3). However, ongoing threats to CMS and Children's Health Insurance Program (CHIP) funding jeopardize child health outcomes. Pediatric health is significantly shaped by social determinants of health (SDoH), encompassing healthcare access and quality, neighborhood and built environment, social and community context, education, and economic stability (1, 4). Children disadvantaged by racism, socioeconomic status, geography, or environment face worse outcomes across these domains.

While health insurance is foundational to access, additional pillars—medical homes, feasibility, and timeliness of care—remain critical (5). Despite Medicaid and Medicare's aims to reduce disparities, gaps persist (6, 7). Rural children face heightened barriers, prompting recommendations for expanded telemedicine and reduced geographic restrictions (8). Among children with autism spectrum disorder (ASD), more generous Medicaid Home and Community-Based Services (HCBS) waivers helped reduce Black–White disparities in unmet needs, whereas private insurance mandates showed no impact (9, 10). Medicaid-insured children with asthma frequently visit emergency departments but often can't afford prescribed medications (11). Marginalized groups—including children of immigrants, those in foster care or with disabilities, and racial, ethnic, and religious minorities—remain disproportionately uninsured and below the poverty line (12). Structural racism and poverty underpin pediatric health inequities within the SDoH framework.

Given the strong evidence linking social determinants of health (SDoH) pediatric health outcomes, several interventions have been developed to reduce or mitigate pediatric health disparities (13–17). CMS has committed to improving pediatric health, minimizing disparities, and lowering costs (18), but faces challenges, including limited and biased data, cultural and language barriers, and gaps in provider awareness (6, 7, 19). To address these barriers, CMS has worked to implement a standardized health equity framework (19).

This commentary examines five published articles, 4 specifically identifying CMS funded interventions addressing pediatric health disparities.

## Current scope of CMS-funded interventions to address health disparities in pediatric populations

The five included articles examined were published between 2013 and 2023. Study designs included a randomized control study (*n* = 1), intervention study (*n* = 1), cross-sectional study (*n* = 1), an innovation program (*n* = 1) and policy statement (*n* = 1). Sample sizes ranged from 129 to 36,000, including low-income families, Medicaid-insured children and adolescents, and Children's Health Insurance Program recipients.

First, a randomized controlled trial in Philadelphia tested Functional Family Therapy (FFT) among 129 adjudicated youth at high risk of gang involvement (6–13, 18–20). The intervention engaged families, educational institutions, juvenile justice, and child welfare systems. Treatment group youth had greater service utilization, lower 18-month recidivism, and reduced costs—primarily by avoiding residential placements—highlighting the value of evidence-based interventions funded through Medicaid.

Second, a 2017 intervention at Cincinnati Children's Hospital involved 36,000 Medicaid-insured children with asthma in Hamilton County, Ohio (21). A hospital-led collaborative focused on long-term asthma management, medication-in-hand strategies, and outpatient care coordination. Within 3 years, asthma-related hospitalizations and ED revisits declined, controller medication access improved, and acute care use decreased among high-risk patients—demonstrating how targeted delivery reforms can reduce utilization and improve outcomes.

Third, a study in Chicago evaluated how neighborhood disorganization affects care engagement among 6,458 children with chronic conditions (22). Children in the most disorganized neighborhoods were significantly less engaged in care, especially Black youth. Older adolescents (14–18 years old) also showed lower engagement than younger children. Employing health workers from participants' communities mitigated medical mistrust, underscoring the role of neighborhood and racial context in access disparities (23–26).

Fourth, Changing High-Risk Asthma in Memphis through Partnership (CHAMP) (27, 28), a risk-based innovation program in Shelby County, TN, serving 1,348 children (90% Black) to improve asthma care through community health workers, social needs screening, and partnerships addressing environmental and social determinants. The predominantly low-income, single-caregiver households faced unstable, hazard-prone housing and frequent relocations, which served as the impetus for the CHAMP study design team to develop targeted mitigation strategies through coordinated medical, community, and legal supports.

Finally, a policy statement assessed the Children's Health Insurance Program (CHIP) and its impact on insurance coverage, access, health status, and care quality for near-poor children nationwide (29). CHIP improved primary and preventive care utilization, but challenges remain in identifying eligible children and maintaining enrollment. State-level variability and the block grant model limit program flexibility. Recommendations include minimizing cost-sharing differences, increasing pediatric provider reimbursement, and expanding eligibility monitoring—especially for youth up to age 26, foster children, and those with undocumented parents.

## Discussion

These interventions underscore how insurance gaps, racial inequities, and neighborhood disadvantage drive pediatric health disparities, while CMS-supported programs offer evidence-informed strategies to address them. Additional CMS initiatives, such as funding Health Care Innovation Awards for high-cost, high-prevalence, and high-severity conditions, have downstream impacts on reducing pediatric health disparities (27). Efforts like the New England Asthma Innovation Collaborative (NEAIC), delivering home-based asthma care to over 1,100 Medicaid- (23) and CHIP-enrolled children through community health workers, have reduced hospitalizations and missed school days, but sustained impact depends on continued CMS investment to avoid erosion of progress and worsening child health outcomes (28).

The overlap in findings and recommendations across these CMS-funded studies highlights actionable strategies to reduce pediatric health disparities. Central to each intervention was federal funding, effective community engagement, including the deployment of trained community health workers (28, 30, 31) and care coordinators, which improved trust and care continuity. Tailored policies—such as providing inhalers at discharge to address pharmacy access barriers—also enhanced outcomes. Insurance expansion emerged as a critical factor in improving access, quality, a outcomes. Medicaid expansion reduced in-hospital mortality and improved access to rehabilitative care, while Children's Health Insurance Program Reauthorization Act (CHIPRA) and the Affordable Care Act (ACA) advanced coverage and delivery for near-poor children (21, 29). Patient education on insurance benefits further increased access among previously uninformed communities.

Importantly, race and residence significantly influenced care engagement; families in disorganized and minority-concentrated neighborhoods were less likely to engage in care (22). These factors are essential for targeting outreach and monitoring eligibility. Finally, health disparities impose substantial financial burdens on families and the healthcare system, costing billions annually (32, 33). Expanding evidence-based, CMS-supported interventions can yield both improved health outcomes and cost savings through reduced utilization and recidivism (21, 32, 33).

## Conclusion

As this commentary illustrates, CMS-funded interventions have demonstrated measurable progress in advancing pediatric health equity through community engagement, targeted service delivery, and expanded insurance coverage. However, persistent disparities—rooted in structural racism, poverty, and geographic inequities—continue to undermine outcomes for marginalized children. While pediatric health research has progressed beyond disparity detection, evidence-based implementation of targeted interventions remains lacking (13). Sustained federal investment in Medicaid and CHIP is essential to preserve and scale proven models of care. Declines in funding threaten not only access to services but also the infrastructure necessary to deliver and evaluate equitable, high-quality care. In a climate of fiscal uncertainty, policymakers must reaffirm their commitment to pediatric populations by protecting and expanding CMS-supported initiatives that address social and structural determinants of health. Without this support, the most vulnerable children face widening health gaps and worse health outcomes.
